# Impact of a school-based intervention on nutritional education and physical activity in primary public schools in Chile (KIND) programme study protocol: cluster randomised controlled trial

**DOI:** 10.1186/s12889-016-3878-z

**Published:** 2016-12-03

**Authors:** Nelly Bustos, Sonia Olivares, Bárbara Leyton, Marcelo Cano, Cecilia Albala

**Affiliations:** Instituto de Nutrición y Tecnología de los Alimentos INTA, de la Universidad de Chile, Santiago, Chile

**Keywords:** Childhood obesity, Food education, Healthy kiosk, Physical activity, Schools

## Abstract

**Background:**

Chile has suffered a fast increase in childhood obesity in the last 10 years. As a result, several school programmes have been implemented, however the effectiveness of these needs to be evaluated to identify and prioritize strategies to curve this trend.

**Methods:**

Cluster randomized controlled trial. Twelve primary public schools chosen at random over three regions of the country will take part in this study. The sample size consisted of a total of 1,655 children. For each region one school will be selected for each of the three nutritional intervention modes and one school will be selected as the control group. The intervention modes consist of the following:

- Healthy Kiosk and nutritional education (KSEAN);
- Optimized physical activity (AFSO);
- Healthy Kiosk and nutritional education (KSEAN) + optimized physical activity (AFSO);
- Control group.

The effectiveness of each intervention will be evaluated by determining the nutritional condition of each child by measuring percentage of body fat, BMI and the z-score of the BMI. This study will also identify the eating behaviours, nutritional knowledge and fitness of each child, along with the effective time of moderate activity during physical education classes.

**Discussion:**

A protocol to evaluate the effectiveness of a school based intervention to control and/or reduce the rates of childhood obesity for children between 6 and 10 years of age was developed. The protocol was developed in line with the Declaration of Helsinski, the Nüremberg Code and the University of Chile Guidelines for ethical committees, and was approved by the INTA, Universidad de Chile ethical committee on Wednesday 12 March 2014.

There is consensus among researchers and health and education personnel that schools are a favourable environment for actions to prevent and/or control childhood obesity. However a lack of evidence on the effectiveness of interventions to date has led some to question the wisdom of allocating resources to programmes. This is the first study of this kind in Chile and could be an important first step to provide guidance to political authorities in relation to which food and nutrition strategies to prioritize to curve this alarming trend.

**Trial registration:**

ISRCTN32136790, registered retrospectively on 05 September 2014.

## Background

Chile has experienced an accelerated process of epidemiologic and nutritional transition. During this process, very rapid changes have taken place, going from a pre-transition condition where infectious maternal and child diseases predominated public health issues during the decade of the 60s, to a post-transitional condition where chronic, non-infectious diseases dominate (ENT) [1]. Studies have identified that this has resulted due to a broader dietary offering, changes in eating patterns and a considerable increase in sedentary behaviours/lifestyle [2, 3].

The Junta de Auxilio Escolar y Becas (JUNAEB), a Chilean government organization, measures the height and weight of all 6-year-old children attending year one of primary school and found that during 2013 the obesity prevalence for this group of children was 25.3% [4].

The World Health Organization (WHO) has indicated that there is convincing evidence that a sedentary lifestyle along with a diet consisting of a high consumption of foods high in calories and deficient in fruits, vegetables, legumes and fat-free dairy products increases the risk of obesity. However, a suitable home and school environment that promotes the choice and consumption of healthy foods could reduce this risk in children [5].

Childhood obesity is one of the main issues affecting public health, not only due to its increased incidence, but also because the obesity is maintained throughout adolescence and adulthood [6–8]. It is associated with a higher risk of cardiovascular diseases [9], diabetes [10], some types of cancer [11, 12], depression [13], discrimination [14] and weight-related problems, as well as other illnesses, where the short- and long-term risks of childhood obesity translate to a decrease in quality of life [15], requiring earlier interventions that result in healthy behaviours within an obesogenic environment [16].

Schools have been identified as a key setting in the reduction or prevention of the prevalence of overweight and obesity [17, 18], as they provide continuous and intensive contact with children during their formative years. The school infrastructure and the physical environment, policies, programmes and staff have great potential to provide a positive influence on the health of children [16]. However, most research has been based on diverse strategies, covering one or more components (nutritional education, reducing time spent in front of the television, providing reading material, modifying the school menu, reducing sedentary behaviours) [19, 20], and has not been able to show convincing results regarding the effectiveness of school-based programmes in reducing overweight and obesity [21].

Even though school-based interventions have been clearly shown to be more effective than interventions in other settings, it has not been demonstrated that interventions consisting of multiple elements are more advantageous than ones consisting of a single element [20, 22, 23]. This has resulted in the growing need to generate high-quality evidence to guide public policymaking in this area.

Since the year 2000, a series of structural and individual initiatives, linked with the promotion of healthy lifestyles to prevent obesity in the population, have been promoted in Chile. These initiatives have implemented programmes to promote healthy lifestyles in schools, such as VIDA CHILE [24] and the Global Strategy against Obesity (EGO-CHILE) [25]. Additionally, in 2012, the National Education Council approved changes to the school curriculum that increased the number of hours dedicated to physical activity in schools to 3 or 4 hours per week, and promulgated a new law, the “Ley de Composición Nutricional de los Alimentos y su Publicidad”, which prohibits the marketing/advertising and the sale inside schools of foods high in calories, saturated fats, sugars and sodium to children under the age of 14 years﻿ [26].

The KIND study looks at addressing the deficiencies of previous studies and as such should enable evaluation of the effectiveness of an integral school-based intervention in diet and physical activity. The study is aimed at controlling the increase in obesity in children aged between 6 and 10 years, from a medium-low and low socio-economic status, that attend public schools. This study will be conducted in three regions of the country and will provide valuable information that should enable the development of an integral view of the dietary and nutritional status of children attending public schools in Chile.

## Methods

### Design

The proposed study is a cluster randomized controlled trial to evaluate the effect of a school-based intervention in nutritional education and physical activity over two school years in children aged between 6 and 10 years, attending primary public schools. Three modes of intervention will be implemented and the results will be measured alongside those of a control group. The intervention modes consist of the following:

- Intervention 1: Healthy Kiosk and nutritional education (KSEAN);
- Intervention 2: Optimized physical activity (AFSO), where the physical education classes will be taken by a specialized physical education teacher or a primary teacher with a specialization in physical education. The effective class time will be a minimum of 70 min, during which half of the time should involve undertaking activities of moderate to vigorous intensity;
- Intervention 3: Healthy kiosk and nutritional education (KSEAN) + Optimized physical activity (AFSO);
- Control group: Physical education classes will follow the curriculum as indicated for the subject of physical education and health (AFS).

The study will be conducted in 12 clusters (schools) in three regions of Chile (VIII, VI and Metropolitan), distributed in four schools per region, randomly assigned to KSEAN, AFSO, KSEAN+ AFSO or control.

#### Study hypotheses

Integrating school-based interventions over a period of two school years that cover nutritional education, along with the implementation of a healthy kiosk and 4 h per week of physical education classes, where 50% of the time involves physical activity of moderate to vigorous intensity, is more effective in controlling obesity in children between 6 and 10 years of age than implementing each of these interventions separately.

Incorporating nutritional education into the curriculum, where the dietary message provided in the “Chilean Dietary Guidelines” [27] published in 2012 is conveyed, along with the implementation of healthy kiosks at schools, improves the dietary knowledge and behaviours of children between 6 and 10 years of age and guides them to choose foods low in calories inside schools, thereby helping to manage the increase in childhood obesity.

Optimized physical education and health classes, i.e. those undertaken by a specialized physical education teacher, with a total duration of 4 hours per week in separate blocks, where 50% of the effective class time involves conducting activities of moderate to vigorous intensity designed to improve the fitness levels of children aged between 6 and 10 years, helps in managing the increase in childhood obesity.

### Cluster inclusion criteria

The inclusion criteria included:

- Primary public schools located within the Metropolitan Region, Region VI and Region VIII of Chile.
- Schools that are classed with a high school Vulnerability Index by JUNAEB (IVE ≥ 60 JUNAEB) [28].
- Schools that have not been included in previous interventions with programmes that promote healthy lifestyles.
- Full-time co-educational schools with a minimum of 600 students.
- Schools that have specialized physical education teachers.

### Participant inclusion criteria

Students attending public primary schools regularly between the ages of six and ten.

### Participant exclusion criteria

Children excluded from physical education classes due to medical reasons.

#### Recruitment of school (clusters)

For the purpose of this effectiveness study, all of the information regarding public schools was made available to the municipal education departments. This did not require any additional effort or resources for the purpose of recruitment.

From a total of 71 full-time public primary schools within the three regions previously indicated (17 in the Metropolitan Region, 29 in Region VI and 25 in Region VIII), schools with an IVE ≤ 60 were excluded, leaving a total of 58 schools available. Then schools that had previously undergone intervention as part of healthy lifestyle programmes were also excluded, leaving 45 schools, of which only 28 were co-educational and had more than 600 students between 6 and 10 years of age.

Teaching staff qualifications at each school were reviewed and only 18 schools had specialized physical education teachers (six in the Metropolitan Region, eight in Region VI and six in Region VIII). From these 18 schools, four schools per region were chosen at random.

#### Recruitment of participants

Selection of potential participants for each intervention mode and for the control group is to be conducted after randomly selecting the four schools per region.

Meetings are to be held with representatives from the education board and the principals from the participating schools to explain in detail the stages of this research study and the activities that will be undertaken. Afterwards, each school is to invite the parents of the potential participants to meet with the research team, who will explain in detail the aim of the research study. The parents will also be provided with an information sheet detailing the nature and importance of this study and indicating explicitly that it is necessary to have the parents’/guardians’ consent before a child can participate in this study.

A phone number will also be provided to the parents to use in case of any doubts or concerns related to this study. The children will be invited to participate in the project at this time. All parents that indicate that they do not want to participate will be thanked for their time and cooperation.

Children older than 8 years of age that are motivated to participate will be invited to sign an agreement, which will later be used to inform the parents and invite them to participate by signing the consent form. Both the agreement and the consent form have been previously approved by the ethics committee of the Nutrition and Food Technology Institute (INTA) from the Universidad de Chile.

Once the consent forms have been signed, a third meeting is to be organized, where the activities related to this study to be conducted during the school year will be described in detail.

Parents will be informed that this study will determine the nutritional status of each child by measuring weight, height, skinfolds and waistline. These measurements only require that the children remove their shoes and any other heavy element that they might be carrying. It will also be indicated that they will need to answer a survey aimed at determining their current dietary behaviours and that the children’s fitness levels, along with the intensity of the activities conducted in the physical education classes, will be determined by conducting the following tests:

1. Grip strength: consists of squeezing a dynamometer with both right and left hands. This instrument measures the grip strength of the child [29].
2. Measuring the distance of a jump done with both feet together and without a run-up [29].
3. Measuring the intensity of the physical activity by using a pedometer [30], which is a portable device used to count steps that will be placed on the waist of children and secured with a belt while they participate in the physical education classes.

It will be indicated to the parents that all of these tests will be conducted under the supervision of a qualified physical education teacher with experience in conducting all of the tests along with a teacher from the school. It will also be explained that all of the tests should take approximately 15 min to complete and that these will be conducted at the beginning and end of the school year during class time. The only measurement that will be taken throughout the whole year is the intensity of the physical education class. The child’s nutritional status will be informed by means of a letter addressed to the parents.

It will also be indicated that the participation in this study is voluntary and that a child can choose to stop participating at any moment. Additionally, it will be made clear that the participation in this study will not incur any cost for the families of the children nor the school, and that all of the information collected will be kept confidential and will only be used for this study.

**Fig. 1:**
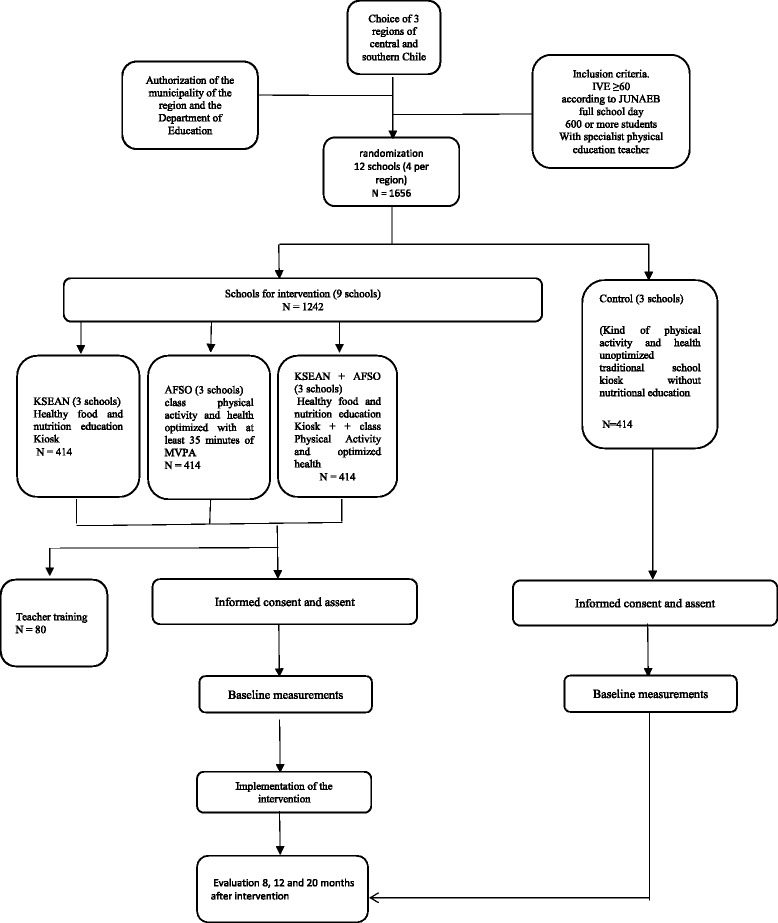
Participant recruitment process

The diagram shown in Fig. 1 below, shows the flow of the suggested recruitment process for this study:

#### Detailed description of the intervention modes

Three modes of intervention and one control group are proposed for this study. The three intervention modes are:

- Intervention 1: Healthy Kiosk and nutritional education (KSEAN);
- Intervention 2: Optimized physical activity (AFSO);
- Intervention 3: Healthy Kiosk and nutritional education (KSEAN) + Optimized physical activity (AFSO).

Each component of the intervention modes is described below:

- Nutritional Education Intervention: This intervention is based on the “Social Cognitive Learning Theory”, which incorporates the interdependency relationship between personal characteristics, behavioural factors and environmental influences [31]. Teachers will provide knowledge and skills in relation to choosing healthy foods at school and at home by providing learning material. Year one to year four students participating in the programme will work with learning material based on the dietary guidelines for the Chilean population that provides nutritional concepts that are reinforced with theory and practical activity, along with the creation of healthy messages [32]. To enhance the effectiveness, the nutritional education activities will be scheduled over 16 sessions of 90 min each. As this is not part of the curriculum, the teacher in charge of each teaching unit (UTP), at each individual school, will define the time when this content will be presented to the students. The implementation of the learning material, including teacher interviews and display of the work conducted by the children in wall displays, will be supervised by a nutritionist, who is part of the research team.
- Healthy Kiosk Implementation: The model described by Bustos and colleagues in the *Manual de Implementación de un Espacio y Punto de Venta Saludable en Escuelas Básicas de Chile* [33] will be replicated in this study. This considers the following:
  The construction of a Healthy Space, located within the school. This space is defined as a place that will encourage healthy lifestyles by promoting recreational activities, the sale of healthy foods and the implementation of various educational strategies aimed at modifying and strengthening healthy behaviour amongst schoolchildren. The Healthy Space will consist of a healthy kiosk, surrounded by tables, chairs and a number of games of an attractive design and painted in bright colours. The kiosk will be built in accordance with what is established in the “Reglamento Sanitario de los Alimentos” [34] and its design will promote the visibility of the food that will be displayed in refrigerated display cases and shelves inside the kiosk.
  The food offering will consist of avocado and fresh cheese or tomato sandwiches, fat-free yoghurt and milk, milk-based desserts, jelly, sugar-free biscuits, cereals, fresh and dried fruits, sugar-free juices and drinks, mineral water and other snacks low in calories. Processed foods will be determined to be low in calories if the nutritional information on the packaging shows the portion sizing and the nutritional information per portion and if this does not exceed 130 Kcal, 3 g of total fats, 20 g of carbohydrates and 140 mg of sodium. For fruits and vegetables a standard portion of 150 g is considered acceptable, as is 30 g for nuts. Also, it will be established that dairy products must be fat-free or low in fat and that other drinks must be sugar-free [33].
  Strategies for promoting the sale of healthy foods and processed foods low in calories will be established based on behavioural economics, which places the food recommended by the Chilean Dietary Guidelines, such as fruits, vegetables and dairy products, amongst others, in the most visible place within the kiosk. Alongside this, positive reinforcement statements promoting the benefits of consuming healthy foods will be displayed to promote their sale. Also, the kiosks will be fitted with an information board displaying various themes related to the promotion of a healthy lifestyle and a price list of foods available for purchase.
  Training will be provided for the kiosk staff in topics such as healthy eating, how to read and understand the nutritional information displayed on food packaging, hygiene, food preparation and handling, and communicational and marketing strategies for promoting the sale of healthy foods.
- Physical Activity Intervention: The optimized physical education classes will consider what is already established in the curriculum for the subject physical education and health, with four teaching hours per week, distributed in blocks of 90 min each on different days of the week. Each school selected for this mode of intervention will have a specialized physical education teacher or a teacher with specific training in physical education. Teachers will receive further training to ensure: 1) that the effective class time has a duration of 70 min; 2) that 50% of the effective time consists of activities that demand a moderate to vigorous intensity. Monitoring will be carried out of all of the physical education classes conducted for years 1 to 4, by external physical education teachers that have been previously trained and standardized. They will measure the effective class time and will select children at random to wear the pedometers during the class.

#### Possible adverse effects of the physical activity intervention

A register will be designed and implemented to identify any potential adverse effect on the children taking part in the intervention that could occur as a result of the increase in physical activity from moderate to vigorous. An injury management protocol will also be designed and provided. With regard to nutritional education, a form will be designed to identify the causes for not complying with the planned activities.

#### Control group

The participants in this group will participate in physical education classes that follow the specifications of the physical education and health curriculum as indicated by the Education Ministry. They will not participate in nutritional education classes and a healthy kiosk will not be implemented at their school. The control group participants will be provided with the same level of support and information, and will be subject to the same evaluations as the participants of the intervention groups in this study.

At the end of the study all of the participants will be invited to take part in nutritional education talks and all participating schools will have the infrastructure of their kiosks repaired. In parallel, teachers from all participating schools will be provided with training in nutritional education and optimized physical education classes.

### Outcome measures

#### Healthy Kiosk with nutritional education and optimized physical activity intervention (KSEAN + AFSO)

##### Primary result variable

Nutritional status of the participants after a 2-year intervention.

##### Operational definition of nutritional status

Obese >2 Z of BMI, Overweight >1 Z of BMI, Normal ≥ − 1 y ≤ 1 Z of BMI.

##### Secondary result variable

Percentage of body fat.

##### Optimized physical activity (AFSO) primary dependent variable

Fitness as measured in the lower body by the distance, in centimetres (cm), of a jump done with both feet together and without a run-up, and in the upper body by a dynamometer, which measures the isometric force in kilograms (kg) of the upper body by participants squeezing the instrument with the right and left hand.

#### Healthy Kiosk with nutritional education intervention (KSEAN)

##### Primary dependent variable

Intake of foods measured as consumed and not consumed.

##### Control variable

Physical activity intensity measured using a pedometer during the optimized physical activity classes.

##### Determination of sample size

The sample size was determined as that required to obtain a standard size of the effect of 0.2 on the BMI z-score, based on a previous nutritional and physical education intervention study conducted in primary schools to prevent childhood obesity by Kain et al. in order to identify this effect in four clusters per intervention mode, with a power of 0.8, a significance of 5% and an inter-cluster coefficient of variance of 0.00012 [35], 140 children per cluster are required along with a minimum of 11 clusters, giving a total of 1540 children. Given that this study was conducted in three regions and that each intervention mode must be conducted in each region, a total of 12 clusters and 1680 subjects are required. Assuming a participation loss of 10% in 2 years of intervention, the total sample size would be 1848 children.

It is anticipated that the grip strength dependent variable will be improved by 20% compared to the study conducted by Rojas C et al. [36]. With the sample size calculated in this manner, in order to have a power of 0.8 at a 95% confidence level, 130 subjects per cluster are required, thereby reaching a total of 1560 children between year 1 and year 4.

### Study schedule

**Fig. 2:**
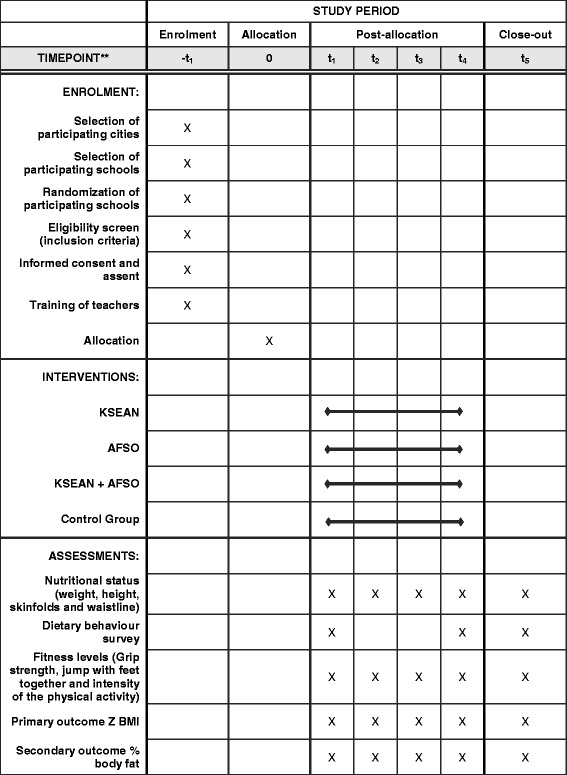
Schedule of enrolment, interventions and assessments

The schedule of enrolment, interventions, and assessments is illustrated in Fig. 2. In this figure time point t_1_ corresponds to the time at which the baseline measurements of all variables will be taken and each intervention mode will commence, this time will be aligned with the beginning of the school year. Time point t_2_ corresponds to the first evaluation after commencing each intervention mode, at this time all variables will be measured on every participating child and will be done 8 months later in line with the end of the school year. Time point t_3_ corresponds to the second evaluation, once again all variables will be measured and this time will correspond with the children returning to school from their summer holidays that is 12 months after time point t_1_. Time point t_4_ corresponds to the final evaluation, where once again all variables will be measured. Time point t_4_ corresponds to the end of the second school year of the intervention that is 20 months after time point t_1_. Final result of the study will be delivered to each school and FOSIS 24 months after commencing the interventions at time point t_5_ (Fig. 2).

### Recruitment status

To obtain a sample size of 1656 children, a total of 1900 needed to be asked to participate in this study. This is based on an estimate of the proportion of children that would potentially satisfy the inclusion criteria and that would be motivated to participate in this study.

Following a review of the sample strategy and the recruitment of the field personnel, the selection of the potential participants commenced on 8^th^ March 2014. By 25^th^ March 2014 a total of 1923 children had been recruited, exceeding the sample size required.

### Data collection

#### Baseline data collection

During the baseline evaluation, participating children will be interviewed by the field personnel, who have been previously trained for this task. The evaluation is to take place at each participating school, following prior authorization by the relevant municipal authorities and school principals.

The following information will be registered.

1. General information about each participant: name, class, gender, RUT (national ID number), and whether the participant receives benefits under the nutritional school programme and/or the social protection programme. These are government programmes that provide benefits to persons, families or territories that are classed as being in a vulnerable situation [37].
2. Anthropometry: weight, height, skinfolds (biceps, triceps, subscapular, suprailiac).
3. Fitness level: dynamometry of right and left hand to measure the isometric strength of the upper body, and length of jump with feet together to measure the explosive strength of the lower body.
4. Duration of moderate to vigorous physical activity during physical education classes.
5. A report of the nutritional knowledge and habits regarding the consumption of dairy products, fruits, vegetables, water, legumes, fish and snacks.
6. A report detailing the main types of food purchased at the school kiosk and the amount of money spent.
7. Food offering at the school kiosk: identification of the types of food and their macronutrient contribution.
8. Number of street food carts/stalls that can be found outside the school and their distance in metres from the main school access.

All of the above evaluations will also be conducted after 8 months of intervention (before starting the summer holidays), after 12 months of intervention (return from summer holidays) and after 20 months of intervention (at the end of the second school year).

### Data handling

#### Data acquisition, management and transfer

All of the participants in this study will be identified by their unique national identification number (RUT). The data collected in the field by the field coordinators will be maintained under confidentiality between the field coordinators and the INTA data coordination centre. A unique identification number will be assigned to each participant once they have been registered in the study and their details have been recorded. This unique identification number will be used from that point forward on all relevant documentation. All participant files will be stored and locked in a secured location. Access to these files will be controlled by the field coordinator.

All of the field data collected will be taken to the INTA on a weekly basis, where they will be validated to identify any inconsistencies and rectify errors. Once validated, the data will be entered into the study database.

The INTA data coordination centre will be responsible for registering all of the data files as they are delivered and notifying the field coordinator if any data are missing. The INTA data coordination centre will also be responsible for the safe keeping of the anonymous and encrypted data.

### Data analysis

The data will be validated by the minimum and maximum values. All the statistical analysis will consider the cluster design, in an intention of treating analysis. Additionally, each intervention protocol will be analysed separately to determine the effectiveness of each intervention.

The normality of the data will be assessed by determining the goodness of fit using the Shapiro-Wilk test, while the homogeneity of the variance will be assessed by using the Bartlett and Levene tests. The results from the Shapiro-Wilk test will be used to describe each variable depending on their normality, variables that are normally distributed will be described by the mean ± SD and range of the 95% CI, while variables that do not follow a normal distribution will be described by their percentile distribution. The results will be shown with their respective 95% confidence intervals.

The difference between each group (intervention mode) will be assessed by conducting parametric tests for normally distributed continuous variables (student *t*-test and ANOVA), and non-parametric tests (Wilcoxon, Kruskal-Wallis and Friedman tests) for non-normally distributed continuous variables. The Chi-square test will be conducted for categorical variables (non-continuous). Logistic regression models will also be developed to analyse the probability of change in the result variables for each intervention mode, controlling by age and gender of the participants and considering the impact of the secondary variables. To evaluate the difference between modes of intervention for continuous dependent variables, a multilevel regression analysis with mixed effects will be conducted to make adjustments by co-variables, as these models do consider the experimental design.

STATA 12 (Copyright 1984–2009 StataCorp) and SAS 9.1 (Copyright (c) 2002–2003 by SAS Institute Inc., Cary, NC, USA) will be used to conduct all of the statistical tests and analysis.

## Conclusion

In relation to the increase in childhood obesity in Chile, there is consensus among researchers, educators and health and education personnel that schools are a favourable environment for actions to prevent and/or control childhood obesity.

Despite the apparent advantages of dealing with obesity in schools, a lack of evidence on the effectiveness of interventions to date has led some to question the wisdom of allocating resources to programmes.

Clearly further studies are required to provide more information on these aspects, and to achieve this, political authorities require specific information on what food and nutrition strategies to prioritize, which, along with physical activity, will provide encouraging results for the control and/or reduction of childhood obesity.

## Abbreviations

AFO: Optimized physical activity
FOSIS: Fund for Solidarity and Social Investment
IC: Confidence interval
ICC: Intracluster correlation coefficient
INTA: Institute of Nutrition and Food Technology
IVE: School Vulnerability Index
JUNAEB: National Board of Student Aid and Scholarships
KSEAN: Healthy food kiosk and nutritional education
MINSAL: Ministry of Health
RSE: Corporate Social Responsibility
SD: Standard Deviation
UTP: Pedagogical Technical Unit

## References

[1] Vio F, Albala C, Kain J (2008). Nutrition transition in Chile revisited: mid-term evaluation of obesity goals for the period 2000–2010. Public Health Nutr.

[2] Kain BJ, Vio DF, Leyton DB, Cerda RR, Olivares CS, Uauy DR (2005). Estrategia de promoción de la salud en escolares de educación básica municipalizada de la comuna de Casablanca, chile. Rev Chil Nutr.

[3] Hawkes C, Smith TG, Jewell J, Wardle J, Hammond RA, Friel S (2015). Smart food policies for obesity prevention. Lancet.

[4] Junaeb. Informe Mapa Nutricional 2013 Departamento de Planificación y Estudios. 2013;1–76. Available from: http://www.junaeb.cl/wp-content/uploads/2013/03/Informe-Mapa-Nutricional-2013.pdf. Accessed 27 Jan 2016.

[5] WHO. Diet, nutrition and the prevention of chronic diseases. World Health Organ Tech Rep Ser. 2003;916:i – viii – 1–149 – backcover. Available from: http://eutils.ncbi.nlm.nih.gov/entrez/eutils/elink.fcgi?dbfrom=pubmed&id=12768890&retmode=ref&cmd=prlinks\npapers3://publication/uuid/734F6B31-260B-4545-A8E4-57F7D35DDEB8 12768890

[6] Ogden CL, Gorber SC, Dommarco J a R, Carroll M, Shields M, Flegal K. Epidemiology of Obesity in Children and Adolescents. Public Health. 2011;69–94. Available from: http://www.springerlink.com/index/10.1007/978-1-4419-6039-9

[7] Biro FM, Wien M (2010). Childhood obesity and adult morbidities. Am J Clin Nutr.

[8] Reilly JJ, Kelly J (2011). Long-term impact of overweight and obesity in childhood and adolescence on morbidity and premature mortality in adulthood: systematic review. Int J Obes.

[9] Raitakari OT, Juonala M, Viikari JSA (2005). Obesity in childhood and vascular changes in adulthood: insights into the Cardiovascular Risk in Young Finns Study. Int J Obes (Lond).

[10] Nguyen NT, Nguyen X-MT, Lane J, Wang P (2011). Relationship between obesity and diabetes in a US adult population: findings from the National Health and Nutrition Examination Survey, 1999–2006. Obes Surg.

[11] Calle EE, Rodriguez C, Walker-Thurmond K, Thun MJ (2003). Overweight, obesity, and mortality from cancer in a prospectively studied cohort of U.S. adults. N Engl J Med.

[12] Vucenik I, Stains JP (2012). Obesity and cancer risk: evidence, mechanisms, and recommendations. Ann N Y Acad Sci.

[13] de Wit L, Luppino F, van Straten A, Penninx B, Zitman F, Cuijpers P (2010). Depression and obesity: a meta-analysis of community-based studies. Psychiatry Res.

[14] Strauss RS, Pollack HA (2003). Social marginalization of overweight children. Arch Pediatr Adolesc Med.

[15] Barajas Gutiérrez MA, Robledo Martín E, Tomás García N, Sanz Cuesta T, García Martin P, Cerrada SI (1998). Calidad de vida relacionada con la salud y obesidad en un centro de atención primaria. Rev Esp Salud Publica.

[16] Katz DL, O’Connell M, Njike VY, Yeh MC, Nawaz H. Strategies for the prevention and control of obesity in the school setting: Systematic review and meta-analysis. Int J Obes. 2008;32(12):1780–9. Available from: http://ovidsp.ovid.com/ovidweb.cgi?T=JS&CSC=Y&NEWS=N&PAGE=fulltext&D=emed8&AN=2008605588\n, http://lshtmsfx.hosted.exlibrisgroup.com/lshtm?sid=OVID:embase&id=pmid:&id=doi:10.1038/ijo.2008.158&issn=0307-0565&isbn=&volume=32&issue=12&spage=1780&pages=1780-1. Accessed 27 Jan 2016. 10.1038/ijo.2008.158 19079319

[17] Institute of Medicine. Preventing Childhood Obesity: Health in the Balance. Washington, DC: The National Academies Press, 2005. doi:10.17226/11015. 22379642

[18] Commission on Ending Childhood Obesity. Report of the Commission on Ending Childhood Obesity. Geneva: World Health Organization, 2016. http://www.who.int/end-childhood-obesity/en/. Accessed 25 Jan 2016.

[19] Xu F, Ware RS, Leslie E, Tse LA, Wang Z, Li J (2015). Effectiveness of a randomized controlled lifestyle intervention to prevent obesity among Chinese primary school students: CLICK-obesity study. PLoS One.

[20] Kriemler S, Zahner L, Schindler C, Meyer U, Hartmann T, Hebestreit H (2010). Effect of school based physical activity programme (KISS) on fitness and adiposity in primary schoolchildren: cluster randomised controlled trial. BMJ.

[21] Connelly JB, Duaso MJ, Butler G (2007). A systematic review of controlled trials of interventions to prevent childhood obesity and overweight: a realistic synthesis of the evidence. Public Health.

[22] Meng L, Xu H, Liu A, van Raaij J, Bemelmans W, Hu X (2013). The costs and cost-effectiveness of a school-based comprehensive intervention study on childhood obesity in China. PLoS One.

[23] Caballero B, Davis SM (2003). CT. Pathways: a school-based, randomized controlled trial for the prevention of obesity in American Indian schoochildren. Am J Clin Nutr.

[24] Salinas J, Cancino A, Pezoa S, Salamanca F (2007). Vida Chile 1998–2006 : resultados y desafíos de la política de promoción de la salud en Chile. Rev Panam Salud Publica.

[25] Ministerio de Salud (2009). Evaluación cualitativa de la implementación de la Estrategia EGO-Escuela.

[26] Ministerio de Salud. Sobre Composición Nutricional de los Alimentos y su Publicidad. 20.606. Recuperado a partir de: http://www.leychile.cl/Navegar?idNorma=1041570.

[27] Ministerio de Salud (2013). Subsecretaria de Salud Publica.

[28] JUNAEB (2005). SINAE Sistema Nacional de Asignación con Equidad para Becas JUNAEB.

[29] Ruiz J. Batería ALPHA-Fitness: test de campo para la evaluación de la condición física relacionada con la salud en niños y adolescentes. Nutr. 2011; Available from: http://search.ebscohost.com/login.aspx?direct=true&profile=ehost&scope=site&authtype=crawler&jrnl=02121611&AN=69682604&h=cxa6b7HUNIaSA68NFyFPF+9ixYAk+N1gsZFTKaTooGeI0e6r4VpEDDhxCDrXlxvFJ8Z+NM44ruMgAKWVIeOz5Q==&crl=c. Accessed 27 Jan 2016. 10.1590/S0212-16112011000600003 22411362

[30] McMinn D, Rowe DA, Stark M, Nicol L (2010). Validity of the New lifestyles NL-1000 accelerometer for measuring time spent in moderate-to-vigorous physical activity in school settings. Meas Phys Educ Exerc Sci.

[31] Bandura A (2000). Health promotion from the perspectives of social cognitive theory.

[32] Bustos N, Benavides C. Qué rico es comer Sano!. Programaciòn de actividades para el fomento de la alimentación saludable y la recreación en niños de 6 a 9 años. Segunda edición 2013.

[33] Bustos N, Kain J, Vio F (2009). Guía para el diseño e implementación de un Espacio y punto de venta Saludable en escuelas básicas de Chile.

[34] Chile. Ministerio de Salud. Reglamento Sanitario De Los Alimentos. D Of la Repub Chile. 2013;40734:24. Available from: http://www.diariooficial.interior.gob.cl/media/2013/12/17/do-20131217.pdf

[35] Kain J, Leyton B, Cerda R, Vio F, Uauy R (2009). 100. Two-year controlled effectiveness trial of a school-based intervention to prevent obesity in Chilean children. Public Health Nutr.

[36] Rojas CJA, del CU VL, Sánchez GV, Banik SD, Argáez SJ (2012). Dinamometria de manos en estudiantes de Merida, México. Rev Chil Nutr.

[37] MIDEPLAN. Conceptos Fundamentales Sistema de protección social Chile Solidario. Ministerio de Planificación y Cooperación. Gobierno de Chile. 2004. Available from: http://www.ministeriodesarrollosocial.gob.cl/admin/docdescargas/centrodoc/centrodoc_170.pdf

